# Research on a Coordination Evaluation and Prediction Model of Water Use and Industrial Ecosystem Development

**DOI:** 10.3390/ijerph20032381

**Published:** 2023-01-29

**Authors:** Jing Wang, Liang Zhang, Huiping Zhang, Ying Zhang

**Affiliations:** 1 School of Economics and Management, Yanshan University, Qinhuangdao 066004, China; 2 Regional Economic Development Research Center, Yanshan University, Qinhuangdao 066004, China; 3 LiRen College, Yanshan University, Qinhuangdao 066004, China

**Keywords:** water resources utilization, industrial ecosystem development, combined empowerment method, BP–CCDM model, GM–BP–CCDM model

## Abstract

Coordinating the relationship between water use and industrial ecosystem development is the key to ensuring high-quality and sustainable development of the industrial economy. In this paper, a model was proposed for evaluating and predicting the coordination between water use and industrial ecosystem development. First, aiming at the coordination of water use and industrial ecosystem development, this paper determined 15 indicators from the aspects of water demand and supply, water conservation and environmental protection, industrial sustainable development, input and output, and industrial development status. The combination weighting method based on game theory was used to determine the weight of the evaluation index. Then, the coordination evaluation model called the back propagation neural network (BP)–coupling coordination degree model (CCDM) and the coordination prediction model called gray models (GM)–BP–CCDM were established. Finally, the model was applied to the coordination evaluation and prediction of water use and industrial ecosystem development in the Hebei Province, China. The results show that the coordination degree of cities in the Hebei Province is moderate. Therefore, based on the research results, some scientific and reasonable suggestions for water resources utilization and industrial ecosystem development were put forward.

## 1. Introduction

Water resources are one of the essential resources for human survival, and the natural resources on which industrial and economic development depends. As one of the world’s major economic powers, the industrial development of China is inseparable from the extensive utilization of water resources. However, in the process of industrial development, the contradiction between the supply and demand of industrial water, serious water pollution, and other problems have resulted in an extremely mismatched and uncoordinated phenomenon in the process of water use and industrial development in China.

With the shortage of water resources becoming a global concern, water resources management has been a hot topic for many scholars. Argyris Panagopoulos argued that the sustainable use of water resources can be achieved through brine management [1]. Ober, J. et al. discussed various activities related to water resource management and analyzed the evaluation of selected quality parameters of tap water in Poland and Ukraine [2]. Dolan, F. et al. evaluated the economic impact of water scarcity in a changing world [3]. Shindhal, T. discussed the most advanced technological and scientific developments in wastewater treatment in the dye industry [4]. Ostad-Ali-Askari, K. et al. argued that consideration of water quality and quantity issues is necessary for the sustainable management of water resources [5]. Jasechko, S. et al. argued that water from groundwater wells can run dry when water tables decline [6]. Zhengtong Li et al. researched various solar technologies for alternative water use in response to the global water crisis [7].

Many scholars have conducted research on the coordination of water resources utilization and industrial structure [8,9]. The main coordination evaluation models and methods used include the coupling coordination degree model [10,11], gray relation and coupling coordination degree model [12], set pair analysis method [13], ecological niche model [14], gray relational degree model [15], water resource ecological footprint model [16], gray relational entropy evaluation method [17], information entropy [18], etc. Some scholars have researched the relationship between secondary industry and water resources utilization. Hu Zhenyun et al. [19] studied the dynamic adjustment of Xinjiang’s industrial structure underwater resource constraints. Jiang Guiqin et al. [20] established a simple coordination evaluation method to validate the coordination of industrial development and water use in the Anhui Province. Zhang Xiaojun et al. [21] studied the adjustment of the secondary industry in Beijing under the constraints of water resources. Guo Jiahang et al. [22] studied the relationship between water use and the development of the secondary industry in Xinjiang.

The industrial ecosystem is a modern industrial development pattern characterized by resource conservation and environmental protection. It is an organic system composed of three subsystems: society, economy, and environment. Many scholars have conducted research on industrial ecosystems [23,24]. Some scholars mainly focused on the diversity of industrial ecosystems [25], the internal control and stability mechanisms of the system [26], and stability evaluation [27]. Shrestha, R. argued for the need to remove and/or recover these toxic, non-biodegradable, and persistent heavy metals from industrial wastewater [28]. Tsujimoto, M. argued that the concept of ecosystems is becoming increasingly important in the field of technology and innovation management [29]. Benitez, G.B. argued that innovation ecosystems allow SMEs to integrate resources and co-create Industry 4.0 solutions [30].

In China, industry is the foundation of the national economy, and its water use problem is the most prominent in agriculture, industry, and services [31]. By studying the problems of water use and the development of industrial ecosystems, regional water use will transition into low consumption and low pollution, and promote the transformation and upgrading of regional industries. The findings will be of interest to those engaged in solving the prominent problems of water use in the process of industrial development and the sustainable development of industrial industry.

Compared with the existing research, the innovation of this paper is reflected in the following aspects: first, from the perspective of industrial water use and industrial ecosystem development, an evaluation index system for the degree of coordination was established. Second, the coupling coordination evaluation model was enriched. The combination weighting method was used for determining the weight of evaluation indicators, and the back propagation neural network evaluation method was adopted for the comprehensive evaluation model. It can not only process a large amount of data quickly and correctly, but also combine the gray prediction model to predict the coordination degree of various cities in Hebei Province. Third, it analyzes the coordination of industrial water use and industrial ecosystem development in various cities in Hebei Province. Some relevant suggestions for the coordinated development of industrial water and industrial ecosystem were put forward. The research results can provide a reference for local government planning.

## 2. Methods

### 2.1. Object of Study

Hebei Province is located in northern China and around Beijing, the political, economic, and cultural center of China. Its geographical location is very important. There are 11 cities in Hebei Province, including Tangshan, Langfang, Shijiazhuang, Baoding, Qinhuangdao, Xingtai, Hengshui, Cangzhou, Zhangjiakou, Handan, and Chengde. There is a serious shortage of water resources in Hebei Province, and the space–time distribution of water resources is uneven. The problem of water pollution is serious, and the demand for water is huge. The contradiction between water supply and water demand is more and more obvious. Therefore, taking 11 cities in Hebei Province as the research objects, this paper studies the coordination between water use and the development of industrial ecosystems in each city in the last 10 years (2011–2020), and forecasts the situation of each city in 2025.

### 2.2. Coordinated Evaluation Index System

**Figure 1:**
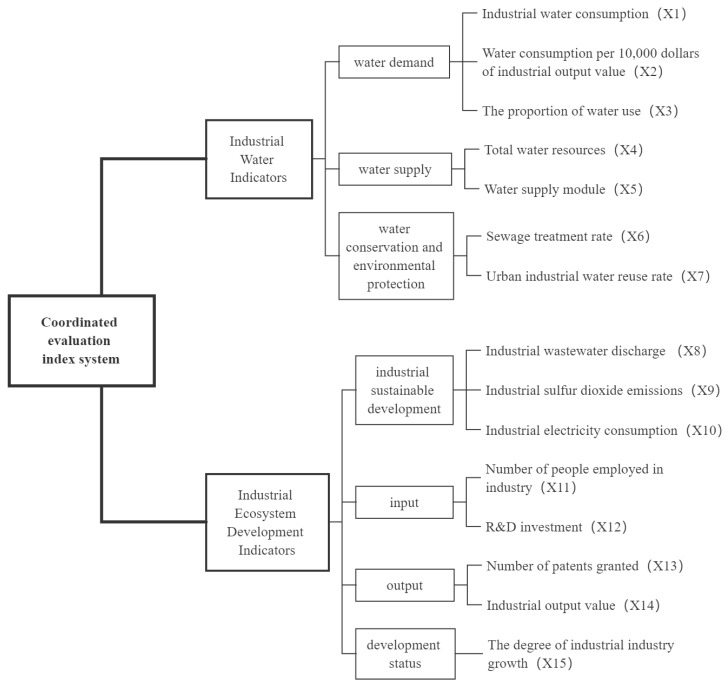
Coordinated evaluation index system.

The evaluation index is one of the indispensable elements in the process of systematic evaluation, and the rationality of its selection is directly related to the accuracy of evaluation results. The principles of scientific nature, comparability, representativeness, and systematization should be considered in establishing a coordinated evaluation index system. Considering that industrial water resource utilization has a prominent contradiction between supply and demand and serious water pollution, we have selected seven evaluation indicators to evaluate water use from three aspects of water demand, supply and conservation, and environmental protection. The basis of industrial development is the need for a certain amount of human, material, and financial input, and industrial output is the basis for measuring industrial development. In addition, the green and sustainable development of industry also needs to be considered. Therefore, we selected eight evaluation indicators to evaluate the development of the industrial ecosystem from the four aspects of industrial sustainable development, input, output, and development status. Regarding the nature of the indicators, except for the six indicators under industrial water demand and sustainable development, which are negative indicators, the rest of the indicators are positive indicators. The specific evaluation index system is shown in Figure 1.

The index of the growth degree of the industrial industry is measured by the elasticity coefficient of the growth of the industrial industry. The calculation method is expressed as follows:

$$X_{15}=\frac{\mathrm{Industrial}\;\mathrm{Industry}\;\mathrm{Output}\;\mathrm{Value}\;\mathrm{Growth}/\mathrm{Industrial}\;\mathrm{Industry}\;\mathrm{Output}\;\mathrm{Value}}{\mathrm{GDP}\;\mathrm{Growth}\;\mathrm{Value}/\mathrm{GDP}\;\mathrm{Value}}$$

Therefore, based on the growth degree of each evaluation object industrial industry, and consulting the opinions of relevant experts in this research field, the scoring standards were established as shown in Table 1.

### 2.3. Coordinated Evaluation Model

To calculate the coupling coordination value of water use and industrial ecosystem development, the coordinated evaluation model is established. Firstly, the combination weighting method combining subjective weighting and objective weighting is adopted to determine the weight of each evaluation index. Then, take the sum of the product of each evaluation index value and the corresponding weight as the expected output value of the back propagation neural network, train the back propagation neural network evaluation model, and bring in the relevant index values to obtain the comprehensive score value. Finally, the coupling coordination model of water use and industrial ecosystem development is established, and the coupling coordination scheduling of each evaluation object is calculated. The model was called BP–CCDM.

#### 2.3.1. Combination Weighting Method Based on Game Theory

The subjective weight of each evaluation index is obtained by constructing the judgment matrix and carrying out the consistency test. The weight obtained by the AHP is recorded as $w_{1i}$.

**Figure 2:**
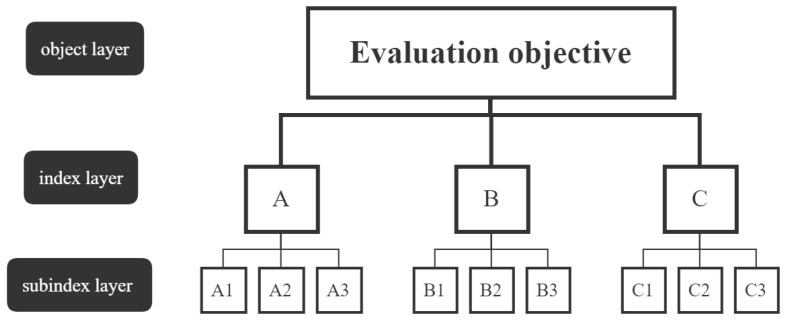
The structure diagram of the AHP hierarchical network.

The AHP method divides complex problems into structures with multiple layers, such as the object layer, index layer, and subindex layer [32]. The structure diagram of the AHP hierarchical network is shown in Figure 2. Index layers and subindex layers include multiple evaluation factors. In pairwise comparisons, a number from 1 to 9 and its reciprocal are used to indicate the relative importance of assessing the factors. The relative importance of the assessment factors is used to establish a consistent judgment matrix. Finally, mathematical theory is applied to calculate the vector corresponding to the maximum eigenvalue of the matrix. Relevant literature was used to determine the weight of AHP methods in evaluation indicators [33,34,35].

The objective weight of the evaluation index is obtained by calculating the coefficient of variation of each evaluation index referring to a related study [36]. The specific formula is expressed as follows:

(1) $$\begin{array}{c} v_{i}=\frac{\delta_{i}}{{\bar{x}}_{i}};w_{2i}=\frac{v_{i}}{\sum_{i=1}^{n}v_{i}} \end{array}$$

where:

- $v_{i}$ is the coefficient of variation for item *i;*
- $\delta_{i}$ represents the standard deviation of item *i;*
- ${\bar{x}}_{i}$ represents the mean value of item *i;*
- n represents the number of evaluation indicators;
- $w_{2i}$ is the objective weight for item *i.*

The combination weighting method based on game theory is used to determine the comprehensive weights. There are several weighting methods, and each weighting method has a corresponding weight set. Then, the linear combination of all weighting methods is shown below referring to a related study [37].

(2) $$\begin{array}{c} w=\overset{L}{\underset{k=1}{\sum }}a_{k}w_{k}^{T}\;\left(a_{k}>0\right) \end{array}$$

(3) $$\begin{array}{c} w_{k}=\left[w_{k1},w_{k2},\ldots ,w_{kn}\right]\;\left(k=1,2,\ldots L\right) \end{array}$$

where:

- $a_{k}$ is the optimized combination of coefficients;
- $w_{k}$ represents a set of weights for each weighting method;
- L represents the number of weighting methods.

Taking the minimization of the dispersion of *w* and $w_{k}$ as the goal, the strategy model is obtained as follows:

(4) $$\begin{array}{c} \min\left\{\overset{L}{\underset{j=1}{\sum }}a_{k}w_{j}^{T}-w_{k}^{T}\right\} \end{array}$$

The optimal first derivative condition of the above equation can be transformed into the following equations:

(5) $$\left[\begin{array}{cccc} w_{1}\cdot w_{1}^{T} & w_{1}\cdot w_{2}^{T} & \ldots & w_{1}\cdot w_{L}^{T}\\ w_{2}\cdot w_{1}^{T} & w_{2}\cdot w_{2}^{T} & \ldots & w_{1}\cdot w_{1}^{T}\\ \vdots & \vdots & \ddots & \vdots \\ w_{L}\cdot w_{1}^{T} & w_{L}\cdot w_{2}^{T} & \ldots & w_{L}\cdot w_{L}^{T} \end{array}\right]\left[\begin{array}{c} a_{1}\\ a_{2}\\ \vdots \\ a_{L} \end{array}\right]=\left[\begin{array}{c} w_{1}\cdot w_{1}^{T}\\ w_{2}\cdot w_{2}^{T}\\ \vdots \\ w_{L}\cdot w_{L}^{T} \end{array}\right]$$

The method of normalizing the calculated optimal combination coefficients is as follows:

(6) $$\begin{array}{c} a_{k}^{*}=\frac{a_{k}}{\sum_{k=1}^{L}a_{k}} \end{array}$$

Finally, the optimized combination weight obtained by the game theory combination weighting method is as follows:

(7) $$\begin{array}{c} w^{*}=\overset{L}{\underset{k=1}{\sum }}a_{k}^{*}w_{k}^{T} \end{array}$$

#### 2.3.2. Back Propagation Neural Network Evaluation Model

Back propagation networks are multi-layer feed-forward neural networks that can implement arbitrary non-linear mapping from input to output. A typical back propagation network is a model consisting of three levels of neurons: an input layer, an output layer, and an intermediate hidden layer [38].

This paper required obtaining two comprehensive score values for water use and industrial ecosystem development, to design back propagation neural network models for training. The establishment process of the back propagation neural network evaluation model mainly includes back propagation neural network structure design, model parameter determination, model training and testing, and model application. The specific content of establishing the back propagation neural network evaluation model is shown in Table 2.

#### 2.3.3. Coupling Coordination Degree Model

On the basis of the training of the back propagation neural network evaluation model, the obtained comprehensive scores of water use and industrial ecosystem development are recorded as *α* and *β*, respectively, and then a coupling coordination degree model is established to evaluate the coordination of water use and industrial ecosystem development.

(8) $$\begin{array}{c} C={\left[\frac{\left(\alpha \cdot \beta \right)}{{\left(\alpha +\beta \right)}^{2}}\right]}^{\frac{1}{2}} \end{array}$$

(9) $$\begin{array}{c} D=\sqrt{C\cdot \frac{\alpha +\beta }{2}} \end{array}$$

where:

- C is the degree of coupling;
- D represents the coupling degree of coordination.

In terms of the coordination grade division of water use and industrial ecosystem development, referring to many studies [10,11,18] on coordination evaluation and considering the characteristics of the coordination evaluation of water use and industrial ecosystem development, this paper divides the coupling coordination degree of water use and industrial ecosystem development into eight grades. The degree is divided into eight grades, and the specific grades are shown in Table 3.

### 2.4. Coordination Prediction Model

In order to obtain the predicted value of the coupling coordination degree of water use and industrial ecosystem development, this paper establishes a coordinated prediction model for water use and industrial ecosystem development based on the GM–BP–CCDM model. The specific prediction model steps are as follows.

First, establish the GM(1,1) gray prediction model to predict the future value of each evaluation index. Accumulate data from the original sequence to obtain a new data sequence. The calculation method of a single datum in the new sequence is as follows:

(10) $$\begin{array}{c} x^{\left(1\right)}\left(t\right)=\overset{t}{\underset{k=1}{\sum }}x^{\left(0\right)}\left(k\right)\;\left(t=1,2,\cdots ,m\right) \end{array}$$

(11) $$\begin{array}{c} x^{\left(0\right)}=\left[x^{\left(0\right)}\left(1\right),x^{\left(0\right)}\left(2\right),\cdots ,x^{\left(0\right)}\left(m\right)\right];\;x^{\left(1\right)}=\left[x^{\left(1\right)}\left(1\right),x^{\left(1\right)}\left(2\right),\cdots ,x^{\left(1\right)}\left(m\right)\right] \end{array}$$

where:

- $x^{\left(0\right)}$ is the original data sequence;
- $x^{\left(1\right)}$ represents the new data series;
- m represents the number of data.

A first-order linear differential equation for $x^{\left(1\right)}\left(t\right)$ is established as follows:

(12) $$\begin{array}{c} \frac{dx^{\left(1\right)}}{dt}+ax^{\left(1\right)}=u \end{array}$$

where:

- a is the development coefficient;
- u represents the gray effect.

Then, solve the gray parameters, and the method is as follows:

(13) $$\begin{array}{c} \hat{a}=\left[\begin{array}{c} a\\ u \end{array}\right]={\left(B^{T}B\right)}^{-1}B^{T}Y_{n} \end{array}$$

where:

- B is the matrix obtained by averaging the accumulated data;
- $Y_{n}$ represents the constant term vector.

Bring the gray parameters into the differential equation to solve, and the results are as follows:

(14) $$\begin{array}{c} {\hat{x}}^{\left(1\right)}\left(t+1\right)=\left[x^{\left(0\right)}\left(1\right)-\frac{u}{a}\right]e^{-at}+\frac{u}{a} \end{array}$$

Discrete and differentiate ${\hat{x}}^{\left(1\right)}\left(t+1\right)$ and ${\hat{x}}^{\left(1\right)}\left(t\right)$ in order to restore the data sequence, and obtain an approximate predicted data sequence as follows:

(15) $$\begin{array}{c} {\hat{x}}^{\left(0\right)}\left(t+1\right)={\hat{x}}^{\left(1\right)}\left(t+1\right)-{\hat{x}}^{\left(1\right)}\left(t\right) \end{array}$$

Then, the established gray model is tested, and the relative error, variance ratio, and small error probability are calculated. If the accuracy of the model meets the requirements, a prediction can be made.

Finally, bring the predicted values into the previously established coordination evaluation model of water use and industrial ecosystem development based on the BP–CCDM model, and calculate the predicted value of the coupling coordination degree of each evaluation object.

## 3. Results

### 3.1. Coordination Evaluation Results

**Figure 3:**
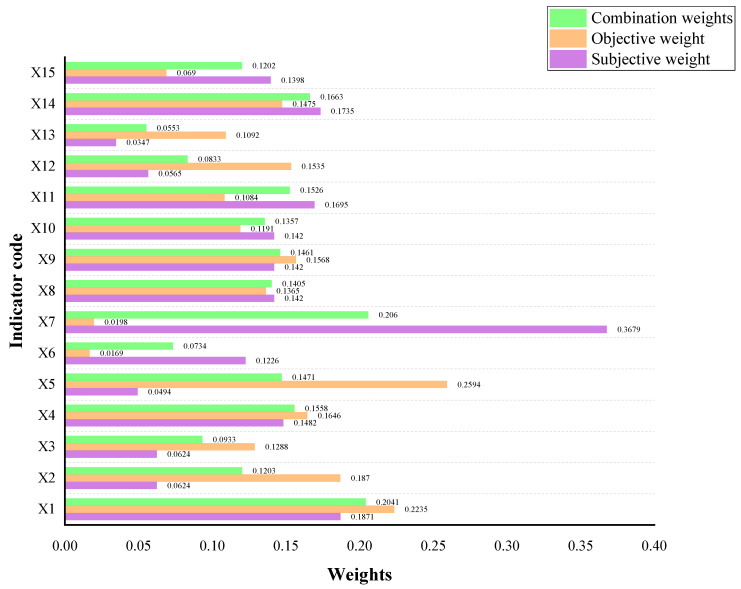
The weight of the evaluation indicator.

In this paper, the subjective and objective weights of the evaluation indicators are obtained by using the AHP subjective weighting and the objective weighting method of the coefficient of variation method. The combination weighting method based on game theory is used to obtain the final weights. The subjective and objective weights and combination weights of the evaluation indicators are shown in Figure 3. 

**Figure 4:**
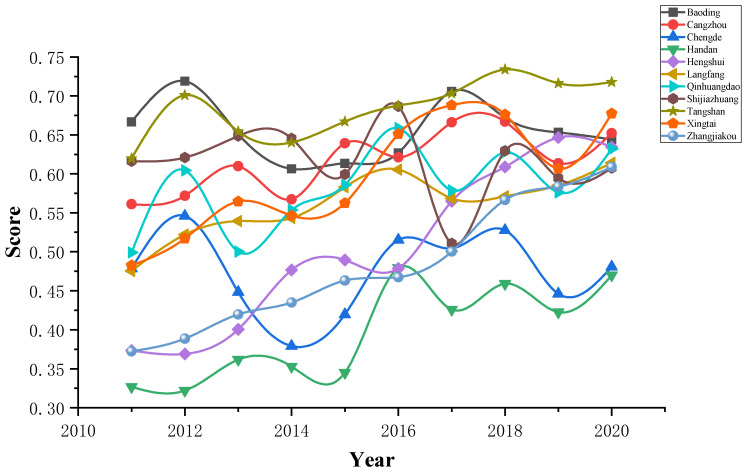
Comprehensive score value of water use.

**Figure 5:**
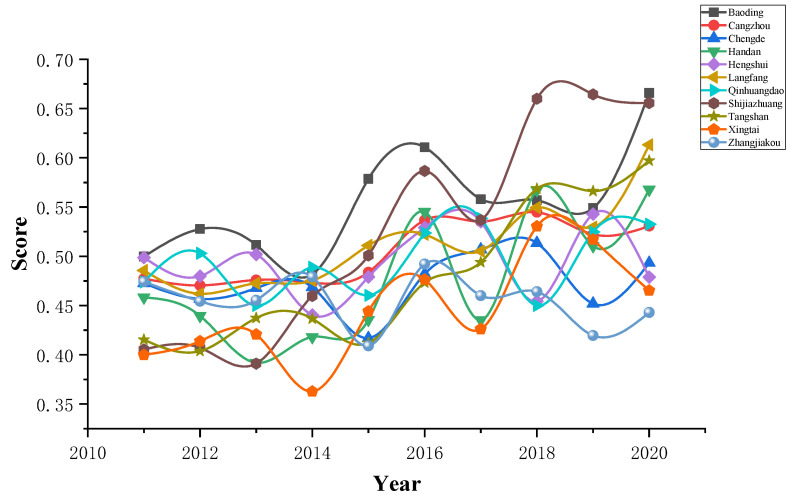
Comprehensive score for industrial ecosystem development.

Firstly, establish the back propagation neural network evaluation model. A total of 88 samples are randomly selected as the training set, and the remaining 22 samples are the test set. Then, calculate the maximum relative error between the actual output value and the expected output value of the test sample, and obtain the maximum relative errors of the water use and industrial ecosystem development evaluation models that are 1.07% and 3.84%, respectively, meeting the accuracy requirement of 5% maximum relative error, which proves that the back propagation neural network model has been trained well. Finally, the comprehensive score of water use and industrial ecosystem development of each assessment object is calculated. The comprehensive scores of water use and industrial ecosystem development are shown in Figure 4 and Figure 5, respectively.

**Figure 6:**
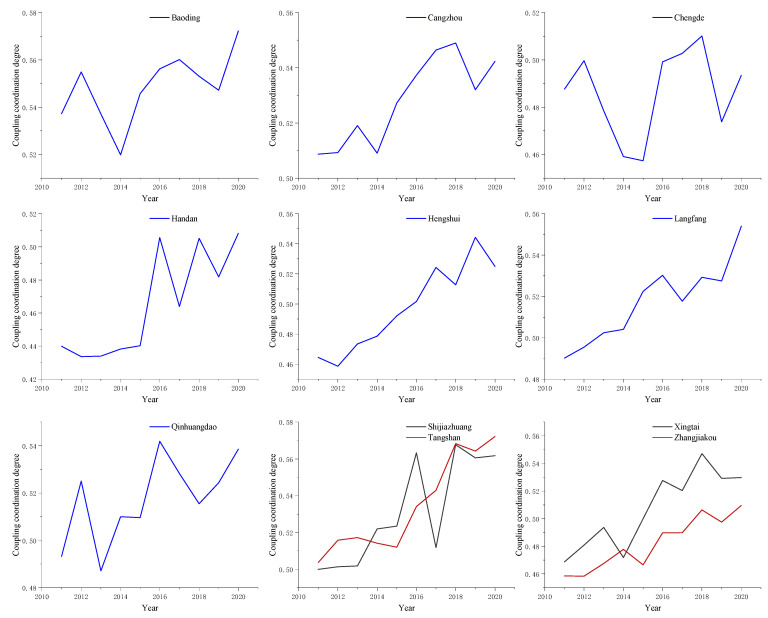
Coupling coordination degree values from 2011 to 2020.

Substitute the comprehensive score value of water use and industrial ecosystem development into the coupling coordination model, and obtain the coupling coordination degree of each evaluation object, as shown in Figure 6.

### 3.2. Coordination Prediction Results

**Figure 7:**
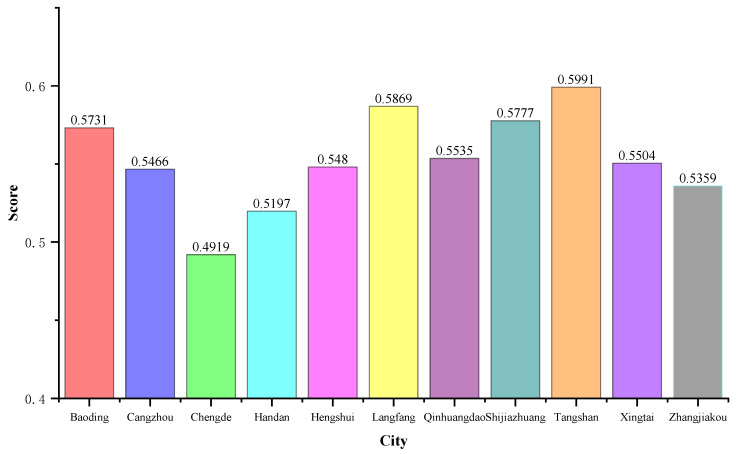
Coordination prediction results for 2025.

The gray prediction model is used to calculate the prediction value of each evaluation index in 2025, and the predicted value is substituted into the BP–CCDM model. Finally, the coupling coordination degree of each city in 2025 is obtained, as shown in Figure 7.

## 4. Discussion

The research results show that the coordination degree between water use and industrial development in various cities of Hebei Province was greatly improved in 2016 compared with 2015. In 2016, the pilot reform of the water resources tax was carried out in Hebei Province, which shows that the reform of the water resources tax has played a certain role in improving the coordination between water use and industrial development in Hebei Province. However, in the years following 2016, the coordination degree of some cities showed a certain degree of fluctuation, which also showed that certain problems were exposed in the water resource tax reform. The reform of the water resources tax is of great significance for enterprises and society with regard to saving water resources and achieving the sustainable development of water resources. Therefore, problems must be found, and solutions must be formulated in time during the reform of the water resources tax. In fact, many problems were exposed after the implementation of the water tax reform in Hebei Province. First, it has caused the problem of increasing the tax burden on urban water supply and public water supply units. Second, there is the problem that it is difficult to collect and manage unlicensed water enterprises. Finally, the price structure of the terminal water supply needs to be adjusted. The emergence of these problems has led to a fluctuating trend in the coordination degree of various cities in Hebei Province after 2016. Particularly, many cities showed a downward trend in 2019. Therefore, in order to improve the coordination degree of cities, the further optimization of water tax reform is one of the key next steps.

According to the coordinated evaluation results of water use and industrial ecosystem development in Hebei Province from 2011 to 2020, it can be seen from the time dimension that the coupling coordination degree of each city has shown a trend of fluctuation and rising over the years, and the range of change is not large. The coupling coordination degree of each city is between 0.4 and 0.6. The coupling coordination degree is on the verge of imbalance or barely coordinated. From the perspective of spatial dimension, except Chengde City, where the coupling coordination degree is fluctuating, the coupling coordination degree of other cities shows an overall upward trend. Baoding, Cangzhou, Langfang, Shijiazhuang, and Tangshan City have a relatively good coupling coordination degree, which is at a barely coordinated degree, while other cities are at a near imbalance or barely coordinated degree. Baoding, Cangzhou, Shijiazhuang, and Tangshan City have better performance in water use, while Baoding Langfang, Shijiazhuang, and Tangshan have performed well in the development of industrial ecosystems. It can be seen that the improvement in coupling coordination degree must be caused by the simultaneous increase in the scores of water use and industrial development, or the increase on one side and the fluctuation trend on the other side.

**Figure 8:**
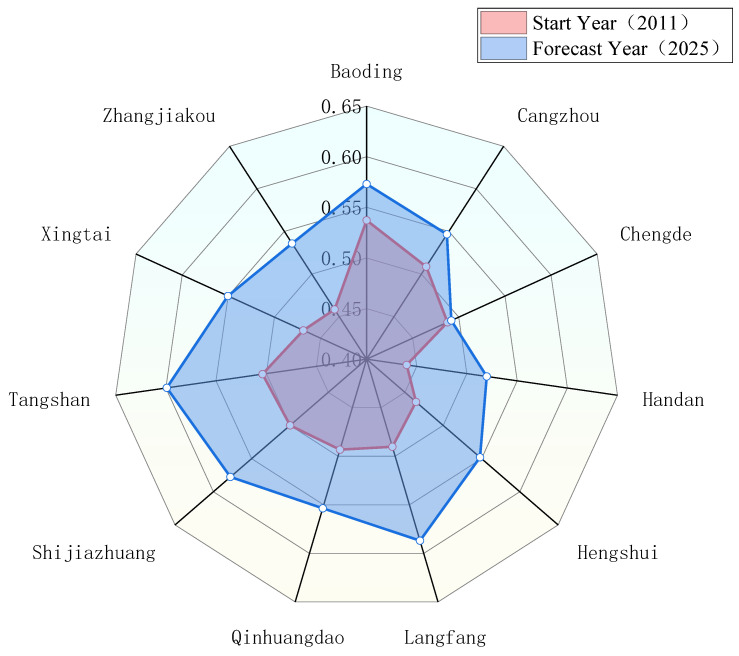
Comparison of coupling coordination degrees between 2011 and 2025.

According to the coordinated forecast results of water use and industrial ecosystem development in Hebei Province, the coupling coordination degree of each city has not changed much compared with 2011–2020. Except for Chengde, which is on the verge of imbalance, other cities have reached a barely coordinated degree. As shown in Figure 8, compared with the start year (2011), the coupling and coordination degree of each city in the forecast year (2025) has improved, and the maximum growth rate of Langfang City is 0.0967. By 2025, the order of coupling coordination degrees of cities from high to low will be Tangshan, Langfang, Shijiazhuang, Baoding, Qinhuangdao, Xingtai, Hengshui, Cangzhou, Zhangjiakou, Handan, and Chengde.

In order to further improve the coordination degree of water use and industrial ecosystem development in various cities, this paper puts forward the following suggestions: From the perspective of water use, the strictest water resource management system should be implemented, and a rigid water resource constraint system should be established. Enterprises that fail to meet the sewage discharge standards should be severely punished, and water-saving actions should be implemented to further control water consumption. At the same time, it is necessary to improve the reuse rate of water resources. From the perspective of industrial ecosystem development, it is necessary to increase the investment in R&D and manufacturing, to encourage innovation to improve the core competitiveness and independent controllability of enterprises, and ultimately achieve high-quality development of industrial enterprises. At the same time, it is necessary to achieve sustainable development and reduce the discharge of wastewater and waste gas. We should also save energy, and the goal is to achieve the green development of industrial enterprises.

## 5. Conclusions

In this paper, the BP–CCDM model provided a complete solution system for the coordinated evaluation of water use and industrial ecosystem development. The GM–BP–CCDM model was established for coordinated evaluation and prediction, which not only ensured the reliability of the evaluation results, but also improved the accuracy of the prediction results. In addition, the model established in this paper can also be used to study other parts of China. This approach can provide solutions to areas with the same problems.

Therefore, the main conclusions of the current study are the following:

First, the coordinated development degree of water use and industrial ecosystem development in various cities was on the verge of imbalance or barely coordinated, and the coupling coordination degree was in a state of slow growth.

Second, according to the results of the coordination evaluation, the coordination degree of cities in Hebei Province in 2016 showed a significant improvement trend compared with 2015, and the coupling coordination degree of some cities showed fluctuations after 2016. 

Third, in order to further improve the coordination between industrial water use and industrial ecosystem development in Hebei Province, the government department must take certain measures.

The selection of evaluation indicators is not perfect. When constructing the evaluation index system of industrial water and industrial development in Hebei Province, there were too few relevant indicators selected due to the difficulty of obtaining relevant data materials. In the future, more indicators could be selected for study when the statistics are more complete.

## Figures and Tables

**Table 1:** Industrial industry growth degree scoring standard.

The Degree of Industrial Industry Growth	Indicator Score
<−10	0
−10~−1	0.15
−1~0	0.35
0~1	0.65
1~10	0.85
>10	1

**Table 2:** Back propagation neural network evaluation model.

Stage	Project	Content
Back Propagation Neural Network Structure Design	input layer	The number of nodes in the input layer of the water use evaluation model is 7; the number of nodes in the input layer of the industrial ecosystem development evaluation model is 8
	output layer	The number of nodes in the output layer is 1, and the expected output value is the sum of the product of the weight of each evaluation index and the standardized value
	hidden layer	The number of hidden layer nodes is based on the combination of empirical formula and trial algorithm
Determination of model parameters	training function	trainlm
	Input-hidden layer transfer function	tansig
	Implicit-output layer transfer function	purelin
	Error function	MSE
	epochs	1000
	lr	0.01
	goal	1 × 10^−7^
Model training and testing	number of training samples	80% of the total sample
	number of test samples	20% of the total sample
Application of the model	model application	The index value of each evaluation object is brought into the trained back propagation neural network evaluation model, and the comprehensive score value of water use and industrial ecosystem development of each evaluation object is calculated, respectively

**Table 3:** Coupling coordination degree classification.

Coupling Degree of Coordination	Degree of Coordination
0~0.2	severe disorder
0.2~0.3	moderately disordered
0.3~0.4	mild disorder
0.4~0.5	on the verge of dysregulation
0.5~0.6	barely coordinated
0.6~0.7	primary coordination
0.7~0.8	intermediate coordinator
0.8~1	highly coordinated

## Data Availability

Data can be made available by the corresponding author upon request.
